# Ginsenoside M1 Induces Apoptosis and Inhibits the Migration of Human Oral Cancer Cells

**DOI:** 10.3390/ijms21249704

**Published:** 2020-12-19

**Authors:** Yu-Chieh Lee, Wei-Ting Wong, Lan-Hui Li, Lichieh Julie Chu, Mridula P. Menon, Chen-Lung Ho, Oleg V. Chernikov, Sheau-Long Lee, Kuo-Feng Hua

**Affiliations:** 1 Department of Biotechnology and Animal Science, National Ilan University, Ilan 260007, Taiwan; lujluj19@gmail.com (Y.-C.L.); non8908@gmail.com (W.-T.W.); mridulamidhu@gmail.com (M.P.M.); 2 Department of Laboratory Medicine, Linsen, Chinese Medicine and Kunming Branch, Taipei City Hospital, Taipei 10844, Taiwan; A1525@tpech.gov.tw; 3 National Defense Medical Center, Department of Pathology, Tri-Service General Hospital, Taipei 11490, Taiwan; 4 Molecular Medicine Research Center, Chang Gung University, Taoyuan 33302, Taiwan; julie.chu@mail.cgu.edu.tw; 5 Liver Research Center, Chang Gung Memorial Hospital at Linkou, Gueishan, Taoyuan 33302, Taiwan; 6 Division of Wood Cellulose, Taiwan Forestry Research Institute, Taipei 100051, Taiwan; chenlung@tfri.gov.tw; 7 G.B. Elyakov Pacific Institute of Bioorganic Chemistry FEB RAS, 690022 Vladivostok, Russia; chernikovoleg@gmail.com; 8 Wellhead Biological Technology Corporation, Taoyuan 325, Taiwan; leesheaulong@gmail.com; 9 Department of Medical Research, China Medical University Hospital, China Medical University, Taichung 406040, Taiwan

**Keywords:** oral squamous cell carcinoma, ginsenoside, biotransformation, apoptosis, migration, caspase, xenograft

## Abstract

Oral squamous cell carcinoma (OSCC) accounts for 5.8% of all malignancies in Taiwan, and the incidence of OSCC is on the rise. OSCC is also a common malignancy worldwide, and the five-year survival rate remains poor. Therefore, new and effective treatments are needed to control OSCC. In the present study, we prepared ginsenoside M1 (20-O-beta-d-glucopyranosyl-20(S)-protopanaxadiol), a major deglycosylated metabolite of ginsenoside, through the biotransformation of *Panax notoginseng* leaves by the fungus SP-LSL-002. We investigated the anti-OSCC activity and associated mechanisms of ginsenoside M1 in vitro and in vivo. We demonstrated that ginsenoside M1 dose-dependently inhibited the viability of human OSCC SAS and OEC-M1 cells. To gain further insight into the mode of action of ginsenoside M1, we demonstrated that ginsenoside M1 increased the expression levels of Bak, Bad, and p53 and induced apoptotic DNA breaks, G1 phase arrest, PI/Annexin V double-positive staining, and caspase-3/9 activation. In addition, we demonstrated that ginsenoside M1 dose-dependently inhibited the colony formation and migration ability of SAS and OEC-M1 cells and reduced the expression of metastasis-related protein vimentin. Furthermore, oral administration or subcutaneous injection of ginsenoside M1 significantly reduced tumor growth in SAS xenograft mice. These results indicate that ginsenoside M1 can be translated into a potential therapeutic against OSCC.

## 1. Introduction

According to the Global Cancer Observatory reports, oral cancer is the sixth most common type of cancer, with an incidence rate of 1.5 per 100,000 inhabitants and a mortality rate of 1.8 per 100,000 inhabitants globally [1]. In 2012, 300,000 oral cancer cases were diagnosed, with 145,000 deaths reported globally [2]. Oral cancer is the leading cause of cancer-related deaths in several countries, such as Taiwan, Sri Lanka, India, Bangladesh, and Pakistan [3]. The most common subtype of oral cancer is the aggressive neoplasm present in oral epithelial cells known as squamous cell carcinoma (OSCC) [4]. Generally, chemotherapy, radiotherapy, and surgery, or a combination of any or all of these therapies, are the treatment options available for oral cancer patients [5,6]. Although these treatment strategies provide temporary relief, they have significant drawbacks in terms of physical, physiological, and financial restrictions on patients [5,7]. In particular, treatment with radiation and chemicals frequently causes severe inflammation and ulceration of the mucous membranes lining the digestive tract and pharynx along with several masticatory disorders, physiological dysfunctions, nausea, and vomiting [5,8]. Better therapeutics are required to meet the medical needs of OSCC patients.

An imbalance in cell proliferation and cell death results in abnormal accumulation of cells, leading to disease and cancer [9]. Programmed cell death, also known as apoptosis, represents a type of cell death featuring numerous unique biochemical and morphological attributes [10]. The caspases that play a significant role in apoptosis have been classified into two main groups, namely, initiator caspases (caspase-2, -8, -9, -10), which initiate the pathway of apoptosis, and effector caspases (caspase-3, -6, -7), which cleave the components of the cell in apoptosis [10]. The activation of caspases is one of the major mechanisms of anticancer drug-mediated apoptosis in cancer cells [11,12]. The BCL-2 family of proteins regulates apoptosis by modulating mitochondrial outer membrane permeabilization, acts as either a death antagonist (e.g., Bcl-2 and Bcl-xL) or agonist (e.g., Bak and Bad), maintains tissue and organ homeostasis by participating in biochemical pathways, and defends against malignant tissue transformation [10]. In addition, the tumor suppressor protein p53 also plays a significant role in the induction of apoptosis and cellular senescence [13].

Ginsenosides, the main active ingredients of ginseng, are known to have a variety of pharmacological activities. Ginsenosides can be metabolized in the body, and a number of recent studies suggest that ginsenoside metabolites, rather than naturally occurring ginsenosides, are readily absorbed in the body and act as active components [14,15]. Ginsenoside M1 (20-O-β-d-glucopyranosyl-20(S)-protopanaxadiol), a major absorbable intestinal bacterial metabolite of ginseng protopanaxadiol saponins, has been demonstrated to induce cell death in many cancers, such as colorectal cancer [16], neuroblastoma [17], lung cancer [18], liver cancer [19], breast cancer [20], and gastric carcinoma [21]. Recently, we demonstrated that ginsenoside M1 ameliorated IgA nephropathy and renal tubulointerstitial lesions in mouse models [22,23]. However, the effect of ginsenoside M1 on human OSCC cells remains unclear. In this study, ginsenoside M1 was evaluated for its anticancer activity in human OSCC cells in vitro and in vivo.

## 2. Results

### 2.1. Preparation of Ginsenoside M1

**Figure 1:**
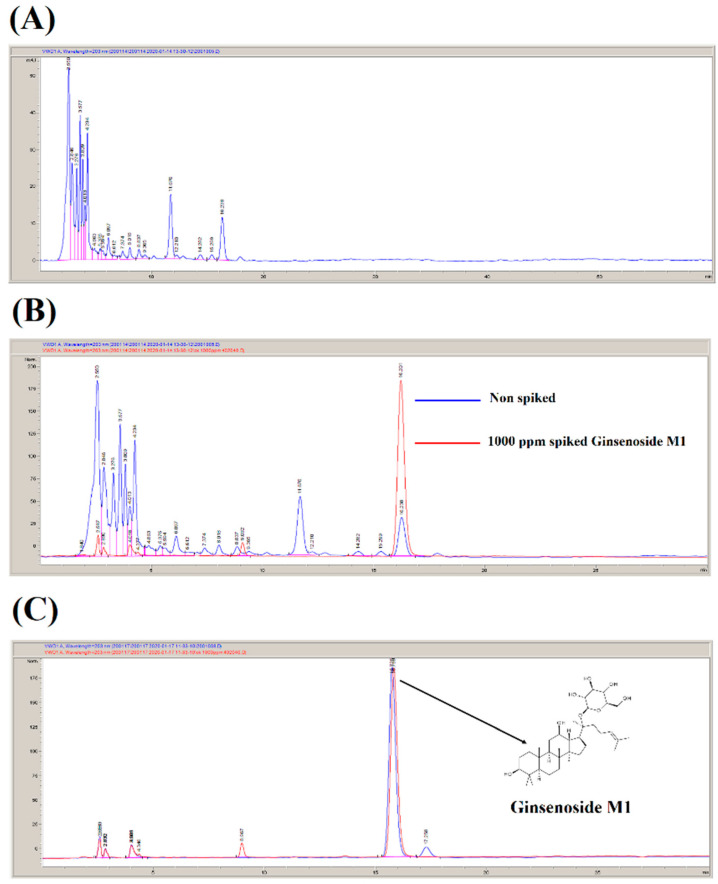
Biotransformation of ginsenoside M1. (**A**) HPLC analysis of *Panax notoginseng* leaves saponins extract. (**B**) HPLC analysis of ginsenoside M1-enriched extract after fungus SP-LSL-002 fermentation. (**C**) HPLC analysis of purified ginsenoside M1.

Ginsenoside M1 (20-O-β-D-glucopyranosyl-20(S)-protopanaxadiol) was prepared according to our US patent (US7932057B2) with some modifications [24]. Briefly, ginsenoside M1 was prepared through the biotransformation of *Panax notoginseng* leaves by the fungus SP-LSL-002 (accession number: BCRC 930079; Food Industry Research and Development Institute, Hsinchu, Taiwan). One kilogram of dry powder of *P. notoginseng* leaves was extracted with 50 L of distilled boiling water for 10 h. The insoluble materials in the extract were removed, and the supernatant was subjected to column chromatography over D101 macroporous resin (Beijing Tong Heng Innovation Technology Co., Ltd., Beijing, China) and washed with reverse osmosis water. The total saponins of *P. notoginseng* leaves were eluted with 95% ethanol and dried under vacuum to give a saponin extract of ca. 200 g (dry weight). The saponin extract was analyzed by HPLC (Agilent, Santa Clara, CA, USA), and the results indicated that the saponin extract contained ginsenosides Rb1, Rb2, Rb3, Rc, and Rd but not M1 (Figure 1A). To produce ginsenoside M1 by the fungus SP-LSL-002 via biotransformation, 100 g saponin extract was mixed with 500 g wheat bran and 1 L distilled water. The mixture was autoclaved (121 °C at 20 psi) for 15 min and inoculated with the fungus SP-LSL-002 (the ratio of the weight of the saponin extract to the weight of the fungus was 1000:1). The fermentation conditions were set at pH 4.5, a temperature of 28 °C, 90% humidity, and a fermentation period 15 days. The fermentation products were then extracted by 75% ethanol, and the extract was dried under vacuum, resulting in a ginsenoside M1-enriched extract of ca. 20 g (dry weight). The ginsenoside M1-enriched extract was analyzed by HPLC, and the results indicated that it contained ca. 20 ± 5% ginsenoside M1 (Figure 1B). The ginsenoside M1-enriched extract was further purified by D101 macroporous resin, anion exchange resin, and reversed-phase chromatography to obtain ca. 2 g ginsenoside M1 with a purity higher than 95% (Figure 1C).

### 2.2. Ginsenoside M1 Induced Human Oral Cancer Cell Death

**Figure 2:**
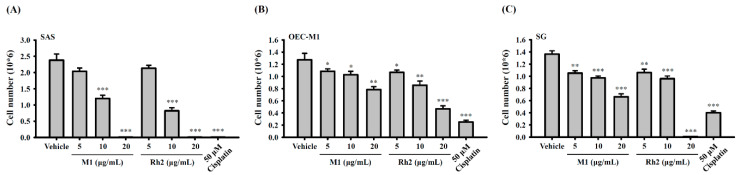
Ginsenoside M1 induced cell death of human oral cancer cells. SAS cells (**A**), OEC-M1 cells (**B**), and SG cells (**C**) were incubated for 24 h with ginsenoside M1, ginsenoside Rh2, cisplatin, or vehicle. The cell numbers were counted by Trypan blue exclusion method. The data were expressed as mean ± SD; *n* = 3. *, **, and *** indicate a significant difference at the level of *p* < 0.05, *p* < 0.01, and *p* < 0.001, respectively, compared to vehicle-control cells. (One-way ANOVA with Dunnett’s multiple comparisons test).

Ginsenoside M1 and ginsenoside Rh2 are the major ginsenoside metabolites with anticancer activities [25]. In addition, ginsenoside M1 and ginsenoside Rh2 are positional isomers [26], therefore, we investigated the effect of ginsenoside M1 and ginsenoside Rh2 in human oral cancer cells. Human oral cancer cells SAS and OEC-M1 were plated at 5 × 10^5^ cells per 6-cm dish in 2 mL of culture medium and were grown overnight at 37 °C in a 5% CO_2_ incubator. The cells were incubated for 24 h with ginsenoside M1 (5–20 µg/mL), ginsenoside Rh2 (5–20 µg/mL), cisplatin (50 µM), or vehicle (DMSO). Each group contained a final DMSO concentration of 0.1%. Thereafter, the cell numbers were counted by the Trypan blue exclusion method. We found that ginsenoside M1 and ginsenoside Rh2 dose-dependently reduced the numbers of SAS cells (Figure 2A) and OEC-M1 cells (Figure 2B), while SAS cells were more susceptible to ginsenoside M1 and ginsenoside Rh2. These results indicated that both ginsenoside M1 and ginsenoside Rh2 induced the death of human oral cancer cells; however, there was no significant difference in the induction of cell death between ginsenoside M1 and ginsenoside Rh2. In addition, we investigated the effect of ginsenoside M1 and ginsenoside Rh2 on SG normal human gingival epithelioid cells. Under the same experimental conditions as in Figure 2A,B, we found that ginsenoside Rh2 at 20 µg/mL completely killed SG cells; however, ginsenoside M1 at 20 µg/mL reduced the cell number by 50% compared to control cells (Figure 2C). These results indicated that ginsenoside M1 was less toxic to SG human normal gingival epithelioid cells than ginsenoside Rh2. Furthermore, cytostatic agent cisplatin significantly reduced the numbers of SAS cells, OEC-M1 cells, and SG cells (Figure 2A–C).

### 2.3. Ginsenoside M1 Induced Apoptosis in Human Oral Cancer Cells

**Figure 3:**
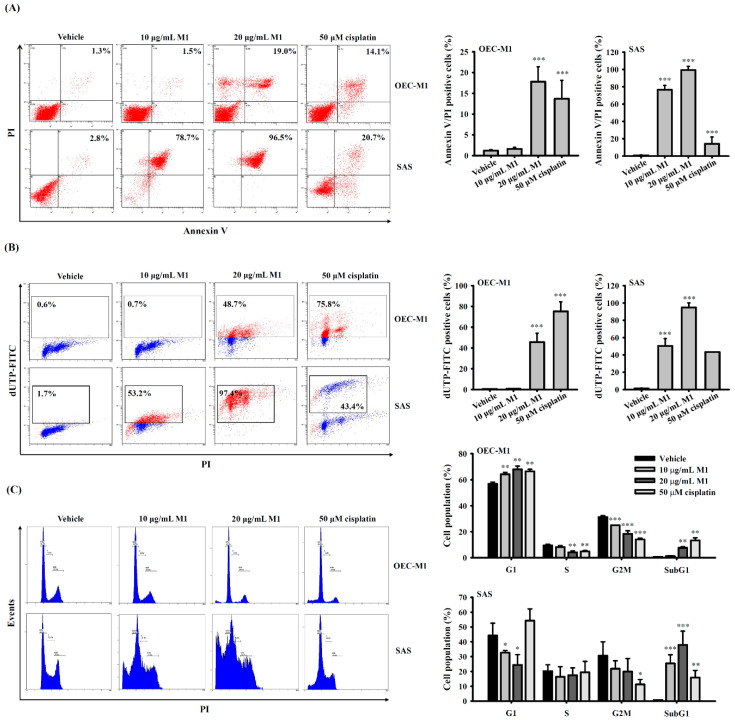
Ginsenoside M1 induced apoptosis in human oral cancer cells. OEC-M1 cells and SAS cells were incubated for 24 h with ginsenoside M1, cisplatin, or vehicle. Apoptosis levels were analyzed by Annexin V/PI double staining (**A**). DNA breaks levels were analyzed by TUNEL assay (**B**). Cell cycle distribution was determined by PI staining (**C**). The data were expressed as mean ± SD; *n* = 3. *, **, and *** indicate a significant difference at the level of *p* < 0.05, *p* < 0.01, and *p* < 0.001, respectively, compared to vehicle-control cells. (One-way ANOVA with Dunnett’s multiple comparisons test).

To gain further insight into the mode of action of ginsenoside M1, we investigated whether ginsenoside M1 induced apoptosis in OEC-M1 cells and SAS cells. The cells were incubated for 24 h with ginsenoside M1, cisplatin, or vehicle. Using an Annexin V/PI double-staining assay, we demonstrated that ginsenoside M1 and cisplatin significantly increased the percentage of OEC-M1 cells and SAS cells with Annexin V/PI double-positive staining, the hallmark of apoptosis, while SAS cells were more susceptible to ginsenoside M1 (Figure 3A). We also demonstrated that ginsenoside M1 and cisplatin treatment for 24 h induced DNA breaks in OEC-M1 cells and SAS cells analyzed by terminal deoxynucleotidyl transferase dUTP nick end labeling (TUNEL) assay, while SAS cells were more susceptible to ginsenoside M1 (Figure 3B). In addition, the effect of ginsenoside M1 on the cell cycle distribution in OEC-M1 cells and SAS cells was determined by flow cytometry after PI staining. We found that the number of OEC-M1 cells in G1 phase increased in a concentration-dependent manner after treatment with ginsenoside M1 and cisplatin for 24 h while the percentage of cells in S and G2/M phase concomitantly decreased compared with the control cells, indicating that ginsenoside M1 and cisplatin induced cell cycle arrest in G1 phase in OEC-M1 cells (Figure 3C). Notably, ginsenoside M1 decreased the percentage of SAS cells in G1 phase, but the percentage of SAS cells in S and G2/M phase were not affected (Figure 3C). Furthermore, the number of OEC-M1 cells and SAS cells in sub-G1 phase increased after treatment with ginsenoside M1 and cisplatin, while SAS cells were more susceptible to ginsenoside M1. These results indicated that ginsenoside M1 induced apoptosis in human oral cancer cells.

### 2.4. Ginsenoside M1 Induced Caspase-Dependent Cell Death

**Figure 4:**
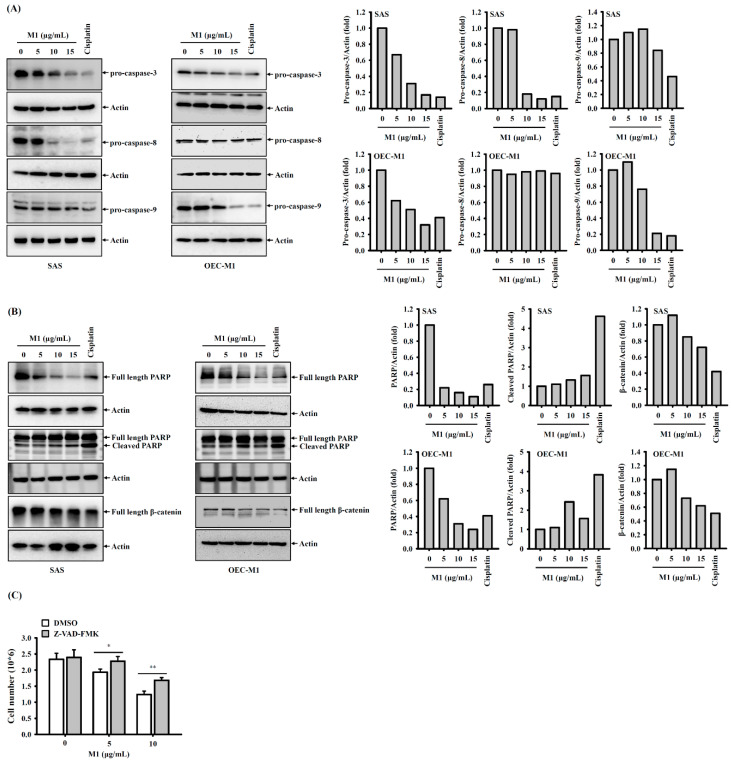
Ginsenoside M1 induced caspase-dependent cell death. (**A**,**B**) SAS cells and OEC-M1 cells were incubated for 24 h with ginsenoside M1, 50 μM cisplatin, or vehicle. The expression levels of caspase 3, caspase-8, caspase-9 (**A**), PARP and β-catenin (**B**) were analyzed by Western blotting. (**C**) SAS cells were incubated for 0.5 h with 20 μM Z-VAD-FMK followed by incubation for 24 h with ginsenoside M1 or vehicle. The cell numbers were counted by Trypan blue exclusion method. The data were expressed as mean ± SD; *n* = 3. * and ** indicate a significant difference at the level of *p* < 0.05 and *p* < 0.01, respectively. (Two-tailed *t*-test).

To investigate whether ginsenoside M1 induced caspase activation, SAS cells and OEC-M1 cells were incubated for 24 h with ginsenoside M1, cisplatin, or vehicle. We found that ginsenoside M1 induced a decrease in the precursors of caspase-3, caspase-8, and caspase-9 in a dose-dependent manner in SAS cells, indicating that caspase-3, caspase-8, and caspase-9 were activated by ginsenoside M1 (Figure 4A). Notably, ginsenoside M1 and cisplatin induced a decrease in the precursors of caspase-3 and caspase-9; however, the precursors of caspase-8 were not affected (Figure 4A). Ginsenoside M1 and cisplatin treatment also induced the degradation of PARP and β-catenin, which were cleaved by active caspase-3 during apoptosis, confirming the caspase-3-activating activity of ginsenoside M1 (Figure 4B). The effect of ginsenoside M1 and cisplatin on PARP was confirmed by detecting the cleaved form of PARP (Figure 4B). To investigate whether ginsenoside-M1-mediated cell death occurred through a caspase-dependent pathway, the effect of the pan-caspase inhibitor Z-VAD-FMK on ginsenoside-M1-mediated cell death was determined. We found that ginsenoside-M1-mediated cell death in SAS cells was reduced by Z-VAD-FMK (Figure 4C), indicating that ginsenoside M1 induced cell death partially through a caspase-dependent pathway.

### 2.5. Ginsenoside M1 Induced the Mitochondrial Death Pathway

**Figure 5:**
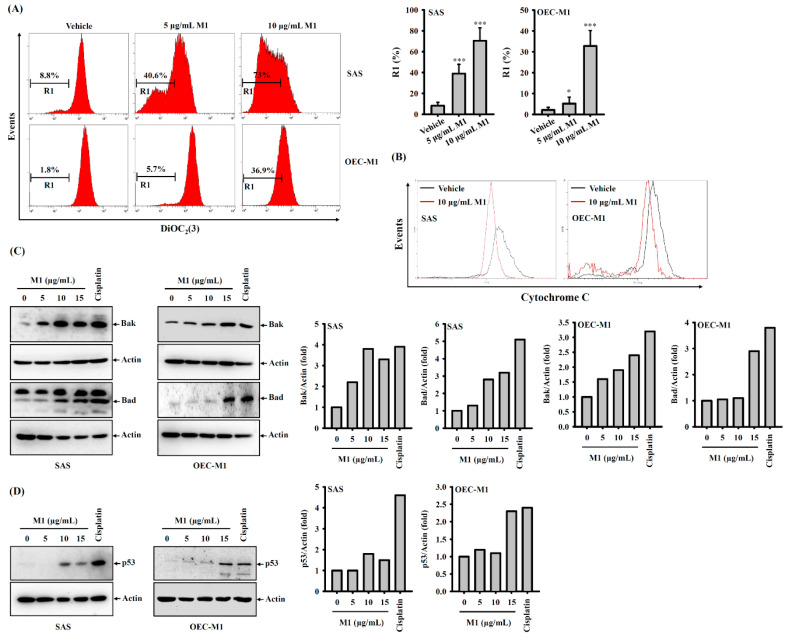
Ginsenoside M1 induced mitochondrial death pathway. SAS cells and OEC-M1 cells were incubated for 24 h with ginsenoside M1, 50 μM cisplatin, or vehicle. The mitochondria membrane potential was analyzed by DiOC_2_(3) staining (**A**). The release of mitochondrial cytochrome c was measured by flow cytometry (**B**). The expression levels of Bak and Bad (**C**) and p53 (**D**) were analyzed by Western blotting. * and *** indicate a significant difference at the level of *p* < 0.05 and *p* < 0.001, respectively, compared to vehicle-control cells. (One-way ANOVA with Dunnett’s multiple comparisons test).

Mitochondria play important roles in apoptotic cell death. Cytochrome c and endonuclease G released from damaged mitochondria activate caspase-9/-3 and induce DNA damage, respectively. We investigated whether ginsenoside M1 treatment induced mitochondrial damage in SAS cells and OEC-M1 cells. We found that ginsenoside M1 treatment induced a reduced fluorescent signal of DiOC_2_(3), a mitochondrial-membrane-potential-sensitive fluorescence dye (Figure 5A). This result indicated that ginsenoside M1 induced mitochondrial membrane potential loss in SAS cells and OEC-M1 cells. Activation of the mitochondrial death pathway can also be identified by the release of mitochondrial cytochrome c. Cytosolic cytochrome c was detected by varying the exposure of SAS cells and OEC-M1 cells to ginsenoside M1 and the level of cytochrome c that remained in the mitochondria was observed to decrease concomitantly (Figure 5B). In addition, the Bcl-2 homologous antagonist killer (Bak) and the Bcl-2-associated death promoter (Bad) proteins are proapoptotic members of the Bcl-2 gene family that are involved in initiating apoptosis. Bak is required to permeabilize the mitochondrial outer membrane during mitochondrial-mediated cell death, and Bad inhibits the antiapoptotic Bcl-2 protein [27]. We found that ginsenoside M1 increased the protein expression of Bak and Bad in SAS cells and OEC-M1 cells (Figure 5C). In addition, we found that ginsenoside M1 increased the expression of p53, an important regulator of DNA damage during apoptosis in SAS cells and OEC-M1 cells (Figure 5D).

### 2.6. Ginsenoside M1 Reduced the Colony-Formation Ability of Human Oral Cancer Cells

**Figure 6:**
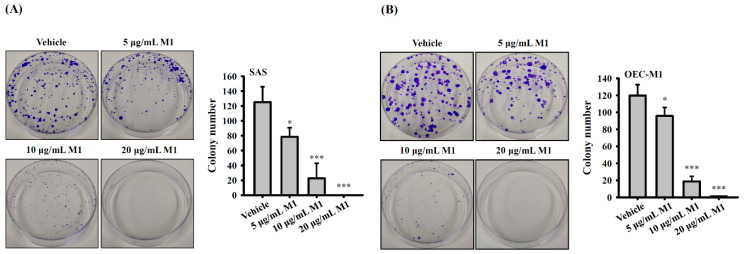
Ginsenoside M1 reduced colony formation ability of human oral cancer cells. SAS cells (**A**) or OEC-M1 cells (**B**) were incubated for 10 days with ginsenoside M1 or vehicle. The colony numbers were analyzed by crystal violet staining. The data were expressed as mean ± SD; *n* = 4. * and *** indicate a significant difference at the level of *p* < 0.05 and *p* < 0.001, respectively, compared to vehicle-control cells. (One-way ANOVA with Dunnett’s multiple comparisons test).

SAS cells or OEC-M1 cells were plated at 150 cells per 6-cm dish in 3 mL of culture medium and were grown overnight at 37 °C in a 5% CO_2_ incubator. The cells were incubated for 10 days with 5–20 µg/mL ginsenoside M1 or vehicle. In SAS cells, 125 ± 20 colonies were formed in vehicle-treated cells, and ginsenoside M1 treatment at 5, 10, and 20 µg/mL reduced the colony number to 78 ± 12, 23 ± 20, and 0 ± 0, respectively (Figure 6A). In OEC-M1 cells, 120 ± 13 colonies were formed in vehicle-treated cells, and ginsenoside M1 treatment at 5, 10, and 20 µg/mL reduced the colony number to 96 ± 10, 19 ± 6, and 1 ± 0, respectively (Figure 6B). These results indicated that ginsenoside M1 dose-dependently inhibited the colony-formation ability of SAS cells and OEC-M1 cells.

### 2.7. Ginsenoside M1 Reduced the Migration Ability of Human Oral Cancer Cells

**Figure 7:**
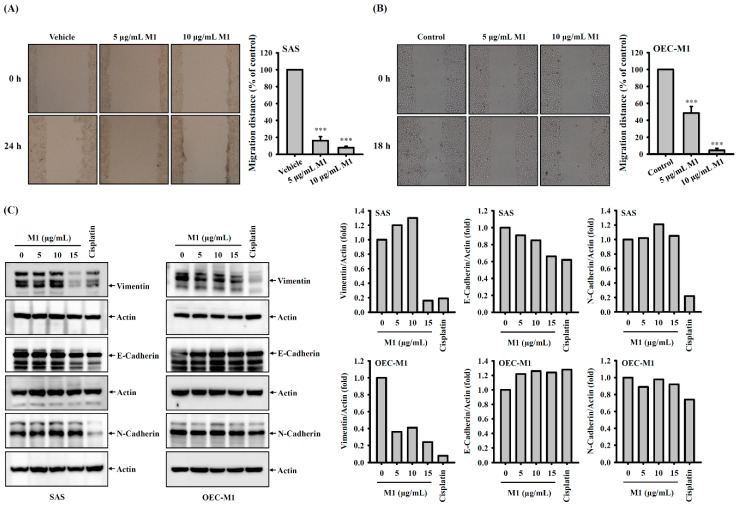
Ginsenoside M1 reduced the migration ability of human oral cancer cells. SAS cells (**A**) or OEC-M1 cells (**B**) were incubated with ginsenoside M1 or vehicle for 24 or 18 h, respectively. The cell migration ability was measured by wound healing assay. (**C**) SAS cells and OEC-M1 cells were incubated for 24 h with ginsenoside M1, 50 μM cisplatin, or vehicle. The expression levels of vimentin, E-cadherin, and N-cadherin were analyzed by Western blotting. The data were expressed as mean ± SD; *n* = 3. *** indicates a significant difference at the level of *p* < 0.001, compared to vehicle-control cells. (One-way ANOVA with Dunnett’s multiple comparisons test).

The cell migration ability was measured by a wound healing assay. Human oral cancer SAS cells or OEC-M1 cells were plated at 5 × 10^6^ cells per 6-cm dish in 2 mL of culture medium and were grown overnight at 37 °C in a 5% CO_2_ incubator until the cells reached 100% confluence and formed a monolayer. We used a sterile 200-μL pipette tip to create a scratched clear zone on the culture dish, and the wounds were photographed by a phase contrast microscope at baseline. The cells were then incubated for 24 h (SAS cells) or 18 h (OEC-M1 cells) with ginsenoside M1 (5–10 µg/mL) or vehicle, and the wounds were photographed again. We found that ginsenoside M1 at 5 or 10 µg/mL significantly inhibited the migration ability of SAS cells to 16.1 ± 4.8 and 7.9 ± 1.6, respectively, compared to vehicle-treated cells (Figure 7A). In addition, ginsenoside M1 at 5 or 10 µg/mL significantly inhibited the migration ability of OEC-M1 cells to 48.5 ± 7.8 and 4.5 ± 2.3, respectively, compared to vehicle-treated cells (Figure 7B). Furthermore, we investigated the effect of ginsenoside M1 on the expression of metastasis-related proteins vimentin, E-cadherin, and N-cadherin in SAS cells and OEC-M1 cells. We found that ginsenoside M1 significantly decreased the expression of vimentin in SAS cells and OEC-M1 cells; however, the expression of E-cadherin and N-cadherin were not affected by ginsenoside M1 (Figure 7C). In addition, cisplatin significantly decreased the expression of vimentin and N-cadherin in SAS cells and OEC-M1 cells, but cisplatin has no effect on E-cadherin expression (Figure 7C).

### 2.8. Ginsenoside M1 Reduced Human Oral Cancer Growth in Mice 

**Figure 8:**
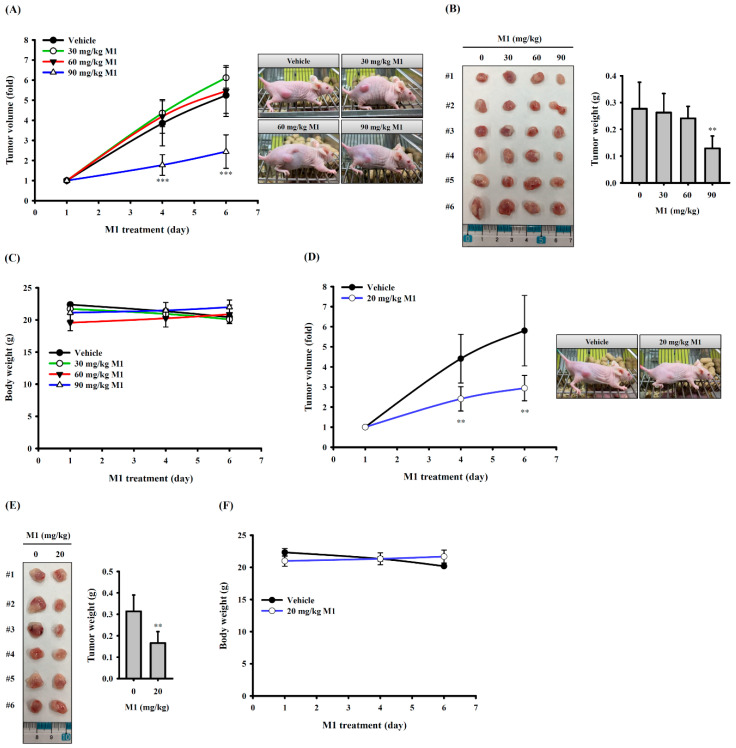
Ginsenoside M1 reduced human oral cancer growth in mice. Tumor volume (**A**), tumor weight (**B**), and body weight (**C**) of mice oral administration of ginsenoside M1 or vehicle. Tumor volume (**D**), tumor weight (**E**), and body weight (**F**) of mice subcutaneous injection of ginsenoside M1 or vehicle. The data were expressed as mean ± SD; *n* = 6. ** and *** indicate a significant difference at the level of *p* < 0.01 and *p* < 0.001, respectively, compared to vehicle-control mice. (One-way ANOVA with Dunnett’s multiple comparisons test).

To evaluate the anticancer activity of ginsenoside M1 in vivo, human oral cancer SAS xenografts were used. We found that oral administration of ginsenoside M1 at 90 mg/kg significantly reduced the tumor size (Figure 8A) and tumor weight (Figure 8B) compared to the vehicle control group; however, oral administration of ginsenoside M1 at 30 and 60 mg/kg did not significantly reduce the tumor size or tumor weight. Oral administration of ginsenoside M1 did not significantly affect the body weight of mice (Figure 8C). In addition, subcutaneous injection of 20 mg/kg ginsenoside M1 also significantly reduced the tumor size (Figure 8D) and tumor weight (Figure 8E) compared to the vehicle control group. Subcutaneous injection of ginsenoside M1 did not significantly affect the body weight of mice (Figure 8F). These results indicated that ginsenoside M1 reduced the growth of human oral cancer in vivo, and no significant adverse effect was observed.

## 3. Discussion

Ginseng is a widely used traditional herbal medicine that promotes health and delays ageing. The main bioactive constituents of ginseng are ginsenosides, which are triterpene saponins [28]. Although a large variety of biological activities of ginsenosides have been demonstrated, orally administered ginsenosides are poorly absorbed [29]. This low absorption in the body limits the biological application of ginsenosides, as the common method of ginseng administration is the oral route. It has been demonstrated that ginsenosides can be metabolized by intestinal microflora and transformed to a deglycosylated form, which has better physiological activities and higher absorption [29]. Ginsenoside M1 is a tetracyclic dammarane-type triterpenoid saponin first identified by Japanese researchers in 1972, and it was demonstrated to be the metabolite of ginsenosides Rb1 and Rb2 by enteric bacteria in the rat large intestine [30]. Scientists are interested in ginsenoside M1 because it has cancer prevention, hepatoprotection, and diabetic improvement effects and exhibits diverse beneficial effects in the immune system, cardiovascular system, and central nervous system [15]. As ginsenoside M1 is a naturally occurring metabolite of ginseng in the body, it is considered safe and might be a potential therapeutic agent for many diseases.

Although the anticancer activity of ginsenoside M1 is well studied, the effect of ginsenoside M1 on oral cancer is still unknown. We demonstrated that ginsenoside M1 reduced the viability of human oral cancer cells mainly by inducing apoptosis in a caspase-dependent manner. Apoptosis induction activity is the major mechanism by which ginsenoside M1 induces cancer cell death. Ginsenoside M1 induced apoptosis in human neuroblastoma SK-N-BE(2) cells [17], human non-small-cell lung cancer A549 and H1975 cells [18], human liver cancer HepG2 and SMMC-7721 cells [19], human breast cancer MCF-7 cells [20], and human gastric carcinoma BGC823 and SGC7901 cells [21]. In addition to apoptosis, ginsenoside M1 also induces autophagy in human liver cancer cells [19], human colon cancer cells [31,32], and human melanoma cells [33]. In human lung and colon cancer cells, ginsenoside M1 mediates apoptosis in an autophagy-dependent manner [18,31,32]; however, the inhibition of autophagy promotes apoptosis in ginsenoside-M1-treated human neuroblastoma cells [17]. Notably, ginsenoside M1 exerts neuroprotective effects by inhibiting autophagy and apoptosis in response to ischemia/reperfusion and oxygen/glucose deprivation and reperfusion injury [34,35]. In our previous studies, we demonstrated that ginsenoside M1 enhanced autophagy and inhibited the inflammatory response in macrophages and in mouse renal tissue [22]. These results suggested that ginsenoside M1 differentially regulates autophagy in a cell type- or stage-dependent manner [17].

The structure of ginsenoside is important for its biological activity. Ginsenoside M1 is a deglycosylated form of Rb1 [30], which induces the apoptosis of human colorectal cancer cells at 30–40 μM; however, Rb1 has no effects on the same cells at concentrations up to 50 μM [36]. In this study we demonstrated that ginsenoside M1 induces the activation of two initiator caspases, caspase-8 and caspase-9 in human oral cancer cells. However, the molecular mechanism of its caspase-8 activating function remains unclear. It has been demonstrated that ginsenoside M1 induced the association of Fas with FasL, FADD and caspase-8 to form death-inducing signaling complex, which subsequently induced the activation of caspase-8 in HeLa cells [37]. Notably, ginsenoside M1-induced caspase-8 activation and apoptosis in HeLa cells were suppressed by cycloheximide, suggesting that these effects were dependent on de novo protein synthesis [37]. In addition, it has been demonstrated that ginsenoside Rh2 activates caspase-8 and induces extrinsic apoptosis through p53-dependent Fas expression in HeLa cells [38]. In our studies we demonstrated that ginsenoside M1 increased the expression of p53; however, the role of p53 on ginsenoside M1-mediated capaspase-8 activation needs further investigation.

In addition, we demonstrated that ginsenoside M1 arrested human oral cancer cells in G1 phase. Although similar effects of ginsenoside M1 on G1 phase arrest and apoptosis were observed in human colorectal and lung cancer cells [18,36], ginsenoside M1 arrested human gastric carcinoma cells in G2 phase [21]. Apoptosis is a highly organized process that is extensively regulated by genes that are associated with cell cycle advancement. Any manipulation of the cell cycle has the potential to either induce or inhibit apoptosis in cells [39]. The oligomerization of the Bak protein in the mitochondrial membrane causes membrane permeabilization, which induces the release of cytochrome c, which in turn results in the activation of the caspase-3 and caspase-9 apoptotic cascades, ultimately causing apoptosis [21]. Our study further pointed to the fact that ginsenoside M1 reduced the mitochondrial membrane potential and increased the expression of p53 and mitochondrial outer membrane permeabilizing Bak protein and apoptosis-promoting Bad protein in oral cancer cells. It is interesting to dissect the role of p53 on ginsenoside M1-mediated Bak and Bad in human oral cancer cells. However, the limitation of this study is not providing the solid evidence showing that ginsenoside M1-mediated Bak and Bad upregulation was dependent on p53 induction. In previous studies p53 has been demonstrated to positively regulate Bak in mouse JB6 epidermis-derived cells [40] and in human non-small-cell lung cancer cells [41]. In addition, p53 activates Bad transcription and expression and forms a p53/Bad complex at the mitochondria to induce apoptosis in human lung adenocarcinoma A549 cells [42]. We suggested that ginsenoside M1 increases Bak and Bad expression in human oral cancer cells may dependent on p53 induction.

Ginsenoside M1 is considered to be a safe ginsenoside metabolite, as it showed selective killing of cancer cells. Ginsenoside M1 induced cell death in human liver cancer cells at IC_50_ values of approximately 40 μM, while it did not significantly decrease the growth of the normal liver cell line L02 [19]. In addition, ginsenoside M1 induced more cell death in human neuroblastoma cells than in normal skin CCD-1079SK and BJ fibroblasts and human umbilical vein endothelial cells [17]. Although ginsenoside M1 also significantly inhibited cell growth in normal human gingival epithelioid SG cells, we demonstrated that ginsenoside M1 was comparatively less toxic towards normal human gingival epithelioid cells than Rh2, a commonly used ginsenoside with anticancer activity [43]. Furthermore, ginsenoside M1 and ginsenoside Rh2 showed different killing effect in normal gingival epithelioid SG cells. As ginsenoside M1 and ginsenoside Rh2 are positional isomers [26], we suggested that the different killing effect of ginsenoside M1 and ginsenoside Rh2 on normal gingival epithelioid SG cells may be structurally selective. Although ginsenoside M1 was less toxic to human gingival epithelioid SG cells than ginsenoside Rh2, ginsenoside M1 showed a similar killing effect in OEC-M1 cells and SG cells. These results suggested that the toxic effect of ginsenoside M1 was not selective to cancer cells, but the side effect may be less than ginsenoside Rh2. However, the detailed mechanism needs to be further investigated. In the 14-day acute toxicity test, ginsenoside M1 administered orally to rats and mice did not cause adverse effects or mortality at the maximum doses of 8 and 10 g/kg, respectively. In the 26-week repeated-dose toxicity study, male rats receiving ginsenoside M1 at a dosage of 120 mg/kg, but not 40 mg/kg, daily transiently developed reversible side effects, including increased focal liver necrosis and plasma ALT/ALP levels, as well as asthenia, hypoactivity, fur loss, and body weight reduction. Notably, these side effects were not observed in female rats receiving ginsenoside M1 at a dose of 120 mg/kg [44]. The different responses in male and female rats need to be further investigated. In our study, the in vivo studies conducted on congenital athymic nude BALB/c mice with xenografts of SAS cells indicated that oral administration of 90 mg/kg or SC injection of 20 mg/kg ginsenoside M1 for five successive days potentially reduced the size of the tumor. Importantly, neither oral administration nor SC injection of ginsenoside M1 reduced the body weight of the mice, and no signs of abnormal clinical toxicity were observed, indicating that there were no significant side effects under these experimental conditions.

Ginsenoside M1 is absent in ginseng, and it is a naturally occurring metabolite of ginseng in the body. As a potential therapeutic agent for many diseases and with high value, there is growing interest in ginsenoside M1 preparation methods [15]. Scientists try to convert ginsenosides into ginsenoside M1 by removing glycosides. Among the variety of deglycosylating methods, biotransformation by microbes or enzymes is the predominant method [45,46]. The production of ginsenoside M1 from ginseng root extract is the most commonly used method; however, the high cost of ginseng root limits this method. In this study, we used *Panax notoginseng* leaves as the ginsenoside source to produce ginsenoside M1 [24], which is a cost-saving method for ginsenoside M1 production. Our studies provide evidence for the ability of ginsenoside M1 to be used as a potential drug against human oral cancers.

## 4. Materials and Methods

### 4.1. Reagents

*P. notoginseng* leaves were purchased from Chinese herbal medicine market (Xi’An, China). Rh2, propidium iodide (PI), cisplatin, Z-VAD-FMK, and crystal violet were purchased from Sigma-Aldrich (St. Louis, MO, USA). Antibodies against Vimentin, E-Cadherin, N-Cadherin, PARP, and Annexin V-FITC Apoptosis Detection Kit were purchased from Abcam (Cambridge, UK). The Apo-direct kit was purchased from BD Bioscience (San Jose, CA, USA). Corning Matrigel Matrix was purchased from Corning Life Sciences (Tewksbury, MA, USA). Antibodies against p53, PARP, Bad, and Bak and actin were obtained from Santa Cruz Biotechnology (Santa Cruz, CA, USA). Antibodies against caspase-3, caspase-8, and caspase-9 were purchased from R&D Systems (Minneapolis, MN, USA). DiOC_2_(3) was purchased from Molecular Probes (Eugene, OR, USA). Antibody against cytochrome c was purchased from Proteintech Group Inc. (Rosemont, IL, USA).

### 4.2. Cell Culture

OEC-M1, SAS, and SG cell lines were provided by Dr. Lichieh Julie Chu of Molecular Medicine Research Center, Chang Gung University, Taoyuan, Taiwan. OEC-M1 cells were cultured in RPMI 1640 medium with 10% heat-inactivated fetal bovine serum (FBS); SAS and SG cells were cultured in MEM-F12 medium with 10% heat-inactivated FBS.

### 4.3. Cell Growth Assay

SAS cells, OEC-M1 cells, or SG cells (5 × 10^5^) were incubated for 24 h with ginsenoside M1, Rh2, or vehicle. The cell numbers were counted by Trypan blue exclusion method.

### 4.4. Cell Cycle Analysis

OEC-M1 cells (1 × 10^6^) were incubated for 24 h with ginsenoside M1, cisplatin, or vehicle, followed by fixed in 70% ethanol on ice for 2 h. The cells were pelleted and washed with phosphate-buffered saline (PBS), followed by resuspended in PBS containing 200 µg/mL RNase and 10 µg/mL PI at 37 °C for 0.5 h. The cell cycle distribution was analyzed by flow cytometer.

### 4.5. DNA Damage Analysis

OEC-M1 cells (1 × 10^6^) were incubated for 24 h with ginsenoside M1, cisplatin, or vehicle, followed by fixed in 70% ethanol on ice for 2 h. The DNA damage was analyzed by the Apo-direct kit. Briefly, the cells were pelleted and washed with PBS and resuspended in 50 µL of the DNA labeling solution (10 µL reaction buffer, 0.75 µL TdT enzyme, 8 µL FITC dUTP, 31.25 µL distilled water) for 1 h at 37 °C. The cells were mixed with 1 mL rinse buffer and pelleted. The cells were resuspended by 1 mL rinse buffer and pelleted again. The cells pellet was resuspended in 0.5 mL PI/RNase staining buffer and incubated in the dark for 0.5 h at room temperature. The cells in PI/RNase solution were analyzed by flow cytometry.

### 4.6. Apoptosis Analysis

OEC-M1 cells (1 × 10^6^) were incubated for 24 h with ginsenoside M1, cisplatin or vehicle. The apoptosis was analyzed by Annexin V-FITC Apoptosis Detection Kit. Briefly, the cells were pelleted and resuspended in 500 µL Annexin V binding buffer. The cells were mixed with 5 µL of Annexin V-FITC and 5 µL PI, and incubated at room temperature for 5 min in the dark. The fluorescent signals of Annexin V and PI were analyzed by flow cytometry.

### 4.7. Detection of Mitochondrial Membrane Potential

A reduction in mitochondrial membrane potential was monitored by DiOC_2_(3) staining as described previously [12]. Briefly, SAS cells and OEC-M1 cells (1 × 10^6^) were incubated for 24 h with ginsenoside M1 or vehicle. Cells were then incubated with 50 nM DiOC_2_(3) for 15 min at 37 °C. Cells were washed three times with PBS and analyzed by flow cytometer.

### 4.8. Detection of Mitochondrial Cytochrome c Release

SAS cells and OEC-M1 cells (1 × 10^6^) were incubated for 24 h with ginsenoside M1 or vehicle. Cells were treated with 100 µL digitonin for 5 min on ice followed by fixed in 4% paraformaldehyde for 20 min at room temperature. After which, the cells were washed three times with PBS, incubated in blocking buffer (3% BSA, 0.05% saponin in PBS) for 1 h, and stained with anti-cytochrome c antibody for 2 h at 4 °C. Thereafter, the cells were further washed three times with PBS and incubated with FITC-labeled secondary antibody for 1 h at room temperature. Cells were washed three times with PBS and analyzed by flow cytometry [11].

### 4.9. Western Blotting

SAS cells (1 × 10^6^) were incubated for 24 h with ginsenoside M1, cisplatin, or vehicle. The cells were washed with ice-cold PBS and lysed with 100 μL ice-cold lysis buffer (20 mM Tris-HCl (pH 7.5), 150 mM NaCl, 1 mM EDTA, 1 mM EGTA, 1% NP-40, 1% sodium deoxycholate, 2.5 mM sodium pyrophosphate, 1 mM β-glycerolphosphate, 1 mM Na3VO4, 1 μg/mL leupeptin, and 1 mM PMSF) on ice for 10 min. The lysates were pelleted and the protein concentrations in supernatants were determined. The protein samples (50 μg each) were subjected to sodium dodecyl sulfate polyacrylamide gel electrophoresis and Western blotting analysis. For quantitating Western blots, the bands intensities from each blot were quantified by densitometric analysis using ImageJ software. The densitometry fold change of each group was calculated by comparing the results with the control group. The band density was normalized to actin before fold change was calculated.

### 4.10. Cell Colony Formation Assay

SAS or OEC-M1 cells (150) were plated in a 6-cm dish for overnight followed by incubated with culture medium containing 5–20 µg/mL ginsenoside M1 or vehicle for an additional 5 days. The culture medium was replaced with a fresh medium containing the same doses of ginsenoside M1 and cultured for an additional 5 days. The cells were washed with PBS and then fixed in a 4% paraformaldehyde for 15 min at room temperature. The cells were stained with 0.1% crystal violet for 0.5 h at room temperature and the colony number was counted.

### 4.11. Cell Migration Assay

The effect of ginsenoside M1 on cell migration was measured by scratch assay. Briefly, SAS cells or OEC-M1 cells (2 × 10^6^) seeded in a 6-cm dish until the cells reached to 100% confluence forming a monolayer, a sterile 200-μL pipette tip was used to create a scratched clear zone on the culture dish. The cells were incubated with ginsenoside M1 for 24 h (SAS cells) or for 18 h (OEC-M1 cells). The wounds were photographed at baseline (before ginsenoside M1 treatment) and 24 h (SAS cells) or 18 h (OEC-M1 cells) after ginsenoside M1 treatment, using a phase contrast microscope.

### 4.12. Antitumor Activity In Vivo

To evaluate the anticancer activity of ginsenoside M1 in vivo, human oral cancer xenografts were used. Six-week-old male congenital athymic BALB/c nude (nu/nu) mice were purchased from BioLASCO Taiwan Co., Ltd. (Ilan, Taiwan), and housed in a room under controlled temperature (23 ± 3 °C) and relative humidity (50 ± 5%). Animal experiments were performed with the approval of the Institutional Animal Care and Use Committee of the National Ilan University (approval number: No. 106-13, 12 April 2017) according to the NIH Guide for the Care and Use of Laboratory Animals. Xenograft mice were established by subcutaneous (SC) injection of 2 × 10^6^ human oral cancer cells SAS (in 75 μL PBS + 75 μL Matrigel) on the backs of the nude mice. After the tumor had reached about 40–60 mm^3^ in size, the mice were randomized into six groups (six mice each): (1) oral vehicle control; (2) oral 30 mg/kg ginsenoside M1; (3) oral 60 mg/kg ginsenoside M1; (4) oral 90 mg/kg ginsenoside M1; (5) SC vehicle control; (6) SC 20 mg/kg ginsenoside M1. The mice were given a daily oral administration or SC injection of either vehicle or ginsenoside M1 for 5 successive days. The mice were scarified at 24 h after receiving the last dose of ginsenoside M1 or vehicle. The tumor volume (TV) was determined by measurement of the length (L) and width (W) of the tumor. The TV on day n (TVn) was calculated as TV (mm^3^) = (L × W^2^)/2. The relative tumor volume on day n (RTVn) versus day 0 was expressed according to the following formula: RTVn = TVn/TV0. The mice body weight was recorded at days 1, 4, and 6 of ginsenoside M1 treatment. The tumors were removed and weighed after the mice were sacrificed.

### 4.13. Statistical Analysis

GraphPad Prism 7.0 software was used for data analysis. Data are shown as mean ± SEM. Statistical significance was determined by *t*-tests (two-tailed) for two groups or ANOVA (with Dunnett’s multiple comparisons test) for three or more groups. *p*-values less than 0.05 were considered to be statistically significant.

## Abbreviations

OSCC	Oral squamous cell carcinoma
TUNEL	Terminal deoxynucleotidyl transferase dUTP nick end labeling
Bak	The Bcl-2 homologous antagonist killer
Bad	The Bcl-2-associated death promoter
PI	Propidium iodide
FBS	Fetal bovine serum
PBS	Phosphate-buffered saline
